# A Novel Trajectory for a Transpedicular Approach in the Treatment of a Highly Downward-Migrated Lumbar Herniation with a Full Endoscopic Technique

**DOI:** 10.3389/fsurg.2022.915052

**Published:** 2022-05-17

**Authors:** Yi Jiang, Rujun Zuo, Shuai Yuan, Jian Li, Chang Liu, Jiexun Zhang, Ming Ma

**Affiliations:** Department of Orthopedics (Minimally Invasive Spine Surgery Branch), Beijing Haidian Hospital (Haidian Section of Peking University Third Hospital), Beijing, China

**Keywords:** transpedicular approach, full endoscopic technique, migrated nucleus, trajectory, lumbar disc herniation

## Abstract

**Background:**

To evaluate the clinical outcome of full endoscopic discectomy using a novel trajectory *via* a transpedicular approach.

**Method:**

Thirty-five patients were enrolled in this retrospective study between July 2014 and October 2019 in the Beijing Haidian Hospital. All patients were treated with full-endoscopic lumbar discectomy *via* a transpedicular approach with an oblique trajectory. The imaging parameters, including pedicle height and angle of trajectory, were recorded. The preoperative and postoperative clinical data were collected for statistical analysis.

**Results:**

All patients underwent successful surgery without severe complications. We compared the visual analogue scale and Oswestry disability index scores before and after surgery. The differences were statistically significant (*p* < 0.05). According to the modified Macnab criteria, the good-to-excellent rate was 85.7% at the last follow-up. The average angles of trajectory in the sagittal and coronal planes were 34.5° ± 2.9° and 47.1° ± 5.0°, respectively.

**Conclusion:**

The new trajectory of the transpedicular approach with a full endoscopic technique for an extremely downward-migrated disc herniation showed excellent results in a small sample study. A precise surgical plan is required, comprising measurements of the pedicle height and angle of the bone tunnel.

## Introduction

The endoscopic spinal technique has achieved satisfactory results in the treatment of lumbar disc herniation (LDH) after continuous development. Currently, the transforaminal and interlaminar approaches are the most widely used approaches. Due to foraminoplasty in the transforaminal procedure, the surgical indications have expanded. However, it still has certain limitations in the treatment of special types of LDH, such as severe prolapse and displacement. In a classification by Lee et al. (1), zone 4, with the disc far downward from the center to the inferior margin of the lower pedicle, was defined as a high-grade inferior-migrated LDH. The likelihood of missing the fragment or disconnecting the stalk is higher in far-migrated discs. Two other studies (2, 3) showed that the incomplete removal of nucleus pulposus was the most important cause of failed percutaneous endoscopic lumbar discectomy. Meanwhile, most researchers (4, 5) suggest that the complete removal of the high-grade inferior-migrated nucleus is still a challenge, because the fragmented free nucleus may increase the residual possibility.

The challenge in removing the highly down-migrated nucleus is due to an occlusion of the pedicle structure. There are some reported methods for overcoming this problem, including the supra-pedicular, contralateral transforaminal, and interlaminar approaches; *via* the adjacent interlaminar approach; dual working channels technology; and the transpedicular approach (3, 6–8). However, these studies are mostly limited as case reports due to a lack of detailed analysis. Gao et al. (5) recommended that the transpedicular approach should be adopted when performing percutaneous endoscopic lumbar discectomy treatment in patients with L5/S1 LDH. Previous studies did not elaborate on this approach and lacked an analysis of its trajectory. In this study, the transpedicular techniques, which can directly expose the migrated nucleus by cutting through a bony channel in the pedicle, will be further expounded.

## Materials and Methods

### Patient Population

Between July 2014 and October 2019, 35 consecutive patients with an extremely downward-migrated LDH were included in this retrospective study. All patients were treated with full-endoscopic lumbar discectomy *via* a transpedicular approach. The inclusion criteria were as follows: (1) unilateral lower limb radiating pain, with or without lower back pain; (2) the lower limb being more painful than the lower back; (3) conservative treatment for 6–8 weeks, which was ineffective; (4) computed tomography (CT) or magnetic resonance imaging (MRI) showed symptoms and signs consistent with the respective segment; (5) MRI sagittal images showed nucleus prolapse to zone 4; and (6) patients who are willing to undergo endoscopic surgery. The exclusion criteria were as follows: (1) imaging data were inconsistent with the patient’s symptoms and signs, and the diagnosis was unclear; (2) severe spinal stenosis; (3) pedicle dysplasia; (4) lumbar instability; (5) the lower pedicle of the surgical segment had pedicle screw fixation; (6) pathological changes such as infection, fracture, or tumor in the responsible segment; and (7) other diseases and inability of the patient to tolerate surgery. Informed written consent was obtained from all patients.

### Surgical Procedure and Postoperative Management

#### Preoperative Planning

(1) By observing the position of the herniated disc and the anatomical relationship with the affected nerve root and the dural sac on CT and MRI axial images, we assessed intervertebral disc calcification, ligamentum flavum hypertrophy, lateral recess stenosis, and facet joint degeneration. The thin-layer scanning MRI sequence of the distal transect for migrated nucleus was recommended to clarify the relationship between the nerve root and the nucleus pulposus. During the measurement of the pedicle height and transverse diameter on CT scan, the transpedicular bony approach needs to meet the placement of the endoscopic working tube. The working channel diameter was 7.5 mm, implying that the pedicle bony channel should be approximately 7–8 mm.
(2) We observed the direction and degree of the migrated disc on the MRI sagittal images and the pedicle morphology on CT sagittal images. To avoid the occurrence of intraoperative fractures, it was recommended that the pedicle height be at least 12 mm for the transpedicular approach.
(3) The pedicle height was measured on the lateral radiograph, and the position of the migrated disc was marked. We observed the structural features of the pedicle.

#### Patient and Medical Team Positioning

The prone and lateral patient positions can be utilized for the surgery. The prone position is recommended because this position is more stable than the lateral position and conducive for the safe application of the dynamic grinding system to treat the pedicle cortex. Simultaneously, it is recommended to bend the knee and hip, because this position can reduce the lumbar lordosis, expand the intervertebral foramen, and relax the exit nerve root. The procedure was performed using dexmedetomidine as sedative and lidocaine as local anesthesia.

#### Portal Design

##### Location

**Figure 1:**
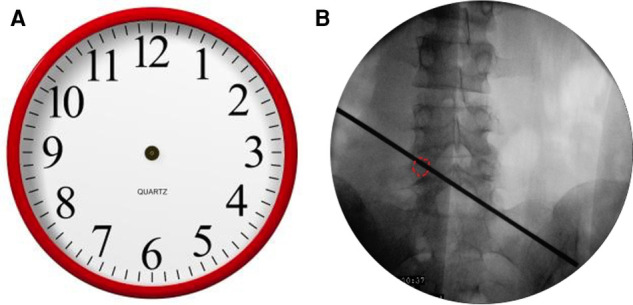
(**A**) The projection of the pedicle in the anteroposterior fluoroscopic view is similar to a clock. (**B**) The puncture direction should be from 11 o’clock to 2 or 3 o’clock on the right pedicle.

The landing point needs to be moved from the superior facet to the pedicle. If the projection of the pedicle in the anteroposterior (AP) fluoroscopic view is similar to a clock (Figure 1A), the landing point is at 11 or 1 o’clock. According to the displacement of the prolapsed nucleus, the puncture direction should be from 11 o’clock to 2 or 3 o’clock on the right pedicle (Figure 1B). On the contralateral left pedicle, the puncture direction should be from 1 o’clock to 10 or 9 o’clock. The distance from the skin puncture point to the midline should be based on the patient’s body type. A previous study (9) suggested that the skin puncture point should be 12 cm away from the midline at L5, 11 cm at L4, and 10 cm at L3.

##### Establishment of the Working Channel

After the administration of local anesthesia and sometimes combined sedation, an 18-gauge needle punctured to the point at 11 or 1 o’clock of the pedicle on the prone position of the patient in AP fluoroscopy, and the needle tip projection in lateral fluoroscopy should be on the posterior part of the pedicle. After accurately positioning the needle, the guide wire and dilators were introduced sequentially. After the soft tissue tunnel was established, the 2.5-mm guide rod with the guide wire was replaced by the 2.5-mm Kirschner wire, which was hammered into the pedicle. The tip of the wire should not enter the median margin of the pedicle. After the Kirschner wire was stably fixed, the 6.5-mm trephine was introduced to cut the bony structure directly. The 7.5-mm trephine was used when necessary. To prevent injury to the dura and nerve root, the reaming should be stopped when it approximates the median pedicle wall, which was monitored with fluoroscopy. The 7.9-mm diameter working tube was inserted after tunnel preparation. The position of the working tube was determined by AP and lateral fluoroscopy. The 6.9-mm-diameter endoscope, which has a 30° angle of view, was introduced in the working tube. During the surgery, continuous irrigation with saline was used.

#### Step-by-Step Description of the Technique(s)

##### Exposure and Observation of the Spinal Canal Structures

**Figure 2:**
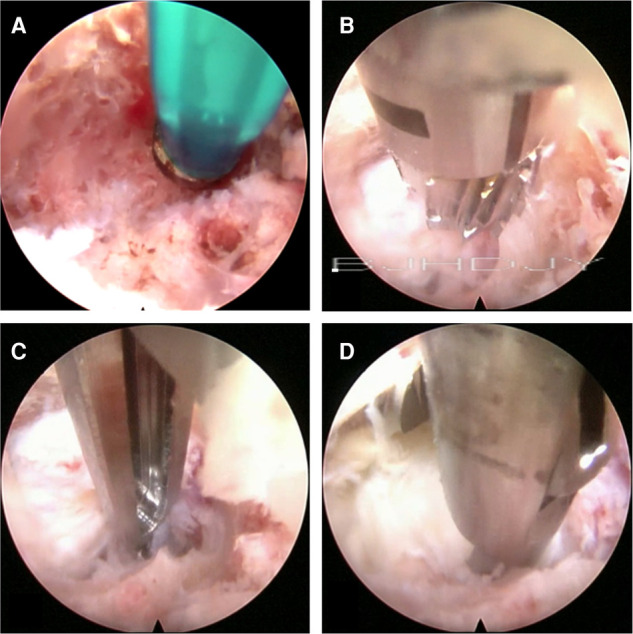
(**A**) Checking the bone of the pedicular medial wall. (**B**) Using the cutting bur to deal with the bone tunnel. (**C**) The Kerrison Rongeur exposes the bone tunnel. (**D**) Removal of the prolapsed nucleus pulposus under the ventral side of the nerve root and dura with the 2.5-mm grasping forceps.

First, the soft tissue was cleaned and the endoscope was placed, after which hemostasis was induced with bipolar radiofrequency. Second, the bone of the pedicular medial wall was probed (Figure 2A). If the medial wall was intact, the dynamic grinding system was used to open the medial wall. The use of steel bur is generally recommended. Although it is more efficient, it may cause bleeding (Figure 2B). There is no ligamentum flavum and less adipose tissue in the lateral recess; therefore, while entering the spinal canal, if the adipose tissue appears, the steel tip grinding drill should be replaced by a diamond bur to reduce bleeding and prevent nerve root injury. The Kerrison rongeur is a good alternative for fenestration to avoid the drill tip entering the spinal canal directly (Figure 2C). Third, after preparing the medial wall of the pedicle, the nerve root and prolapsed nucleus pulposus will appear directly. If the prolapsed nucleus is inferior to the posterior longitudinal ligament, the ligament was opened.

##### Resection of the Prolapsed Nucleus Pulposus

**Figure 3:**
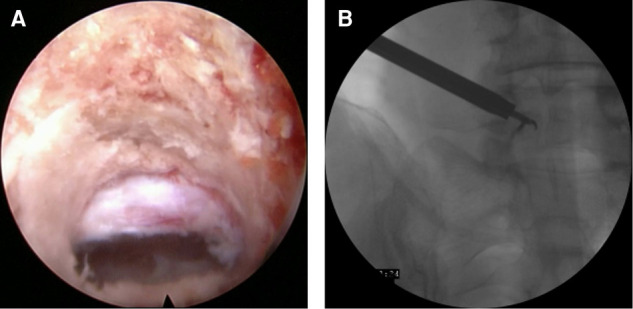
(**A**) The bone tunnel and released nerve root. (**B**) The anteroposterior fluoroscopy to verify the position of the equipment.

After exploring the nerve root and prolapsed nucleus pulposus with bipolar radiofrequency and nerve probe, the prolapsed nucleus pulposus was resected under the ventral side of the nerve root and dura with a 2.5-mm grasping forceps and a semiflexible grasping forceps (Figure 2D). It should be noted that the ganglia structures can sometimes be observed using this approach; therefore, the surgeon should avoid irritation and injury of the ganglia. After complete decompression, the free nerve root and pure bony tunnel were observed on endoscopy (Figure 3A). During the surgery, in case of concern regarding the prolapsed nucleus pulposus residue, the AP fluoroscopy was used to verify the position of the equipment (Figure 3B).

##### Before Completing the Surgery

After resection of the prolapsed nucleus pulposus, it was carefully explored to avoid nucleus pulposus residue. Bone surface bleeding can be stopped using bone wax. Before completing the surgery, we spoke to the patient to determine whether the symptoms had disappeared. If necessary, the straight leg raising (SLR) test was performed intraoperatively. When the patient’s symptoms disappeared, the SLR test was negative, nerve root activity was good, and the endoscope and the working tube were removed. The surgical incision was covered with a sterile dressing after suturing, and drainage was not needed.

#### Complications and Management

Pedicle fracture can be avoided by adopting the following precautions: the diameter of the tunnel should not exceed 8 mm; when the endoscope is inside the tunnel, movements of the system are not recommended; and although it is possible to make the tunnel with a trephine, the use of an endoscopic bone drill under direct endoscopic visualization is highly recommended. Bone bleeding during the drilling of the pedicle can be significant and challenging to stop with the use of a radiofrequency probe. Therefore, we recommend increasing the pressure of continuous saline irrigation and the use of hemostatic agents. Hemostasis, after removing the herniated disc, should be confirmed meticulously to avoid epidural hematoma.

#### Postoperative Care

Patients can rise from the bed four hours after the surgery. Wearing a lumbar brace for protection while sitting and walking is recommended for 6 weeks after the surgery. Non-steroidal drugs can help relieve pain caused by local inflammation in the early stage. We encourage patients to rise from the bed and to do basic functional exercises early.

### Outcome Measurement

**Figure 4:**
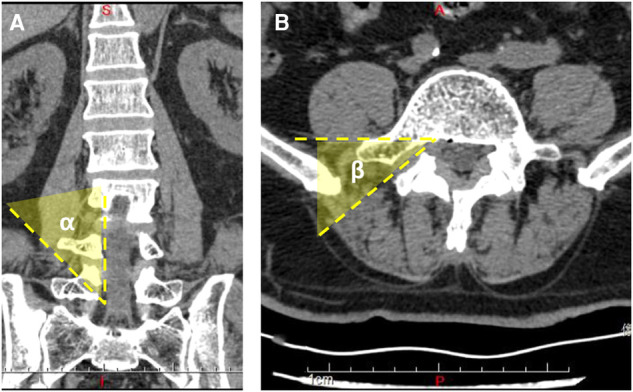
(**A**) Three-dimensional computed tomography scan shows the angle of trajectory in the coronal plane. (**B**) Three-dimensional computed tomography scan shows the angle of trajectory in the sagittal plane.

Demographic data included sex, age, and segment involved in the surgery. The clinical data included visual analogue scale (VAS) for back and leg pain and the Oswestry disability index (ODI) for functional status. The pedicle height was measured using CT and radiography before the surgery, and the angles of trajectory were measured using CT scan postoperatively (Figures 4A,B). The ∠*α* is the angle from the bone tunnel to the medial wall of the pedicle on a sagittal section. The ∠*β* is the angle from the bone tunnel to the posterior vertebral wall on a cross section. The modified Macnab criteria were used for satisfaction assessment in the final follow-up. All patients were followed up for over 2 years.

### Statistical Analysis

The clinical results were analyzed using SPSS version 22 (IBM, Armonk, USA) software. The mean outcome scores (mean ± standard deviation) of pre- and postoperative variables were compared using repeated measures analysis of variance. *p* < 0.05 was considered statistically significant.

## Results

**Figure 5:**
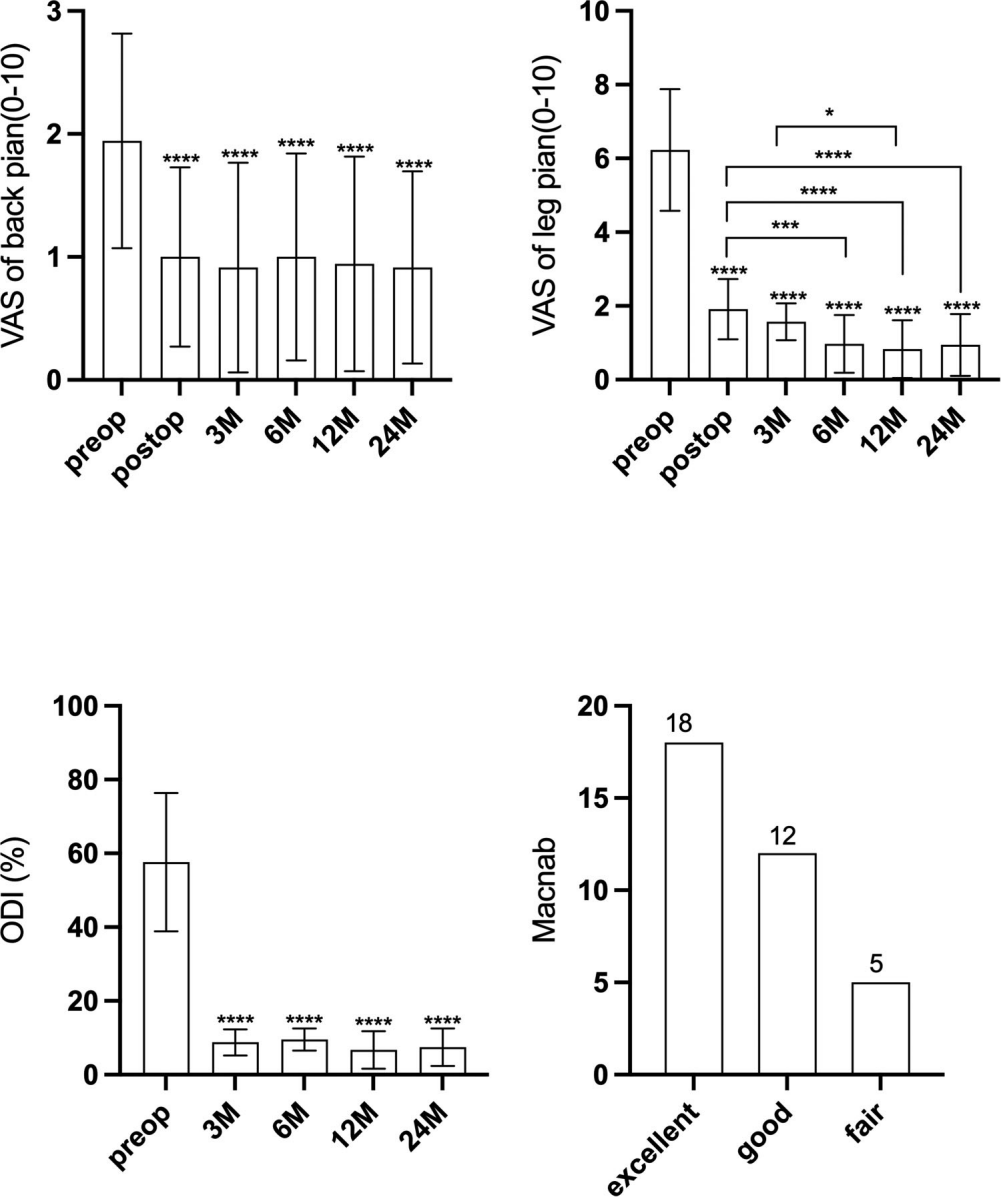
(**A**) The visual analogue scale score of back pain at different intervals. (**B**) The visual analogue scale score of leg pain at different intervals. (**C**) The Oswestry disability index score at different intervals. (**D**) The Macnab criteria at last follow-up.

All the procedures were successfully performed without converting to open surgery. The segmental level was L1/2 in 1 case, L2/3 in 1 case, L3/4 in 4 cases, L4/L5 in 27 cases, and L5/S1 in 2 cases. The mean age of patients was 50.7 ± 10.1 years. The mean duration of surgery was 67.7 ± 12.5 min. The mean preoperative VAS of back pain score was 1.9 ± 0.9, which improved to 0.9 ± 0.8, 1.0 ± 0.7, 1.0 ± 0.8, 0.9 ± 0.9, and 0.9 ± 0.8 at post surgery, 3 months, 6 months, 12 months, and 24 months after surgery, respectively (Figure 5A). The mean preoperative VAS of leg pain score was 6.2 ± 1.6, which improved to 1.9 ± 0.8, 1.6 ± 0.5, 1.0 ± 0.8, 0.8 ± 0.9, and 0.9 ± 0.8 at post surgery, 3 months, 6 months, 12 months, and 24 months after surgery, respectively (*p* < 0.05) (Figure 5B). The VAS of leg pain showed further improvement at 6 months after surgery compared with that at post surgery. The ODI improved from 57.6 ± 18.8 preoperatively to 7.5 ± 5.0 at the final follow-up (*p* < 0.05) (Figure 5C). The VAS and ODI scores significantly improved at each postoperative time point. The good-to-excellent rate in patients was 85.7% (30/35); 18 reported excellent results, 12 reported good results, 5 evaluated their results as fair, and none reported a poor outcome (Figure 5D).

**Figure 6:**
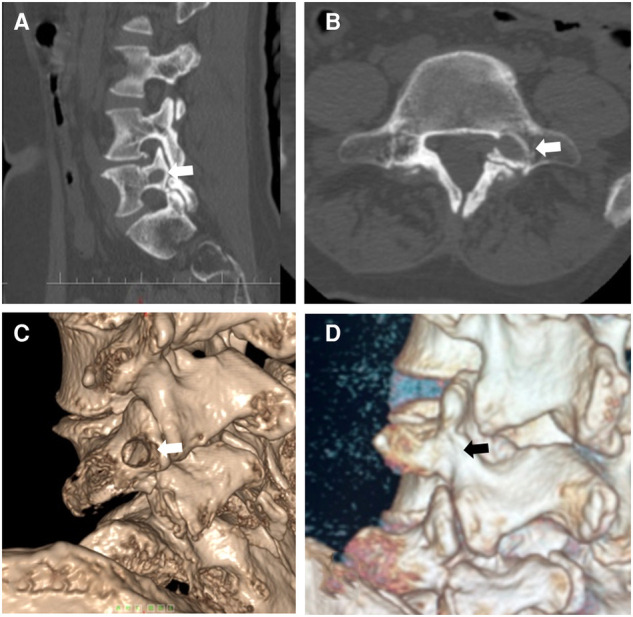
(**A**) Postoperative computed tomography shows a bone tunnel in the sagittal plane. (**B**) Postoperative computed tomography shows a bone tunnel in the horizontal plane.

For the imaging parameters, the mean angles of bone tunnel trajectory were 34.5° ± 2.9° (∠*α*) and 47.1° ± 5.0° (∠*β*), respectively. The mean value of the pedicle height was 12.8 ± 1.1 mm. The mean follow-up was at 42.6 ± 12.6 months. The bone tunnel was found on postoperative CT scan, and no pedicle fractures were observed in the cohort (Figures 6A,B). During the follow-up at 6 months, the hole was healed in all patients compared with that in the postoperative CT scan (Figures 6C,D).

### Typical Case

**Figure 7:**
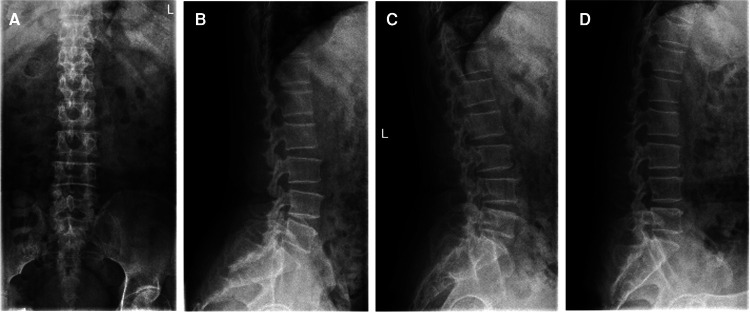
(**A**) The anteroposterior view of the radiograph shows no scoliosis. (**B**) The lateral view of the radiograph shows no spondylolisthesis. (**C**) The extension view of the radiograph shows no instability. (**D**) The flexion view of the radiograph shows no instability.

**Figure 8:**
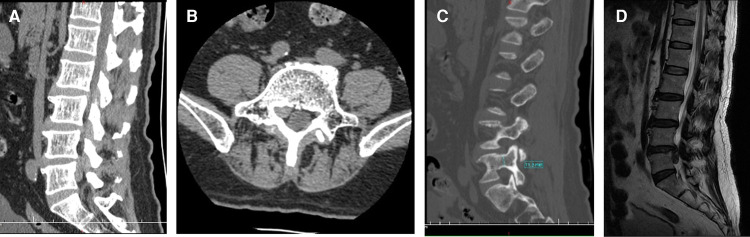
(**A**) Computed tomography scan shows that the distal end of the prolapsed nucleus pulposus is downward beyond the inferior margin of the L5 pedicle. (**B**) Computed tomography scan shows the migrated nucleus that compressed the nerve root. (**C**) The measurement of the pedicle height is 11 mm. (**D**) The magnetic resonance imaging shows that the migrated herniation can be classified into type 4 according to Lee’s classification.

A 66-year-old man presented with lumbosacral pain that had been ongoing for 2 years, which developed to aggravating pain accompanied by radiation pain and numbness in the right lower extremity for over 3 months. Conservative treatment failed. The patient was in a wheelchair when he was admitted to the ward. There was no deformity of the spine, the muscles of all extremities had no atrophy or hypertrophy, and lumbar movement was limited. Interspinous tenderness was found at the L4/5 level, and dorsiflexion myodynamia of the right first toe was of grade II. The rest of the bilateral extremity muscles were normal. The skin sensation of both lower extremities was normal, except the skin at the lateral right calf, lateral right ankle, and dorsum of the right foot. The SLR test was positive (45°) for the right leg. The femoral nerve stretching test was negative. The knee and Achilles tendon reflexes were normal. The VAS score of the back was 3; the VAS score of the right leg was 8; and the ODI score was 80%. Preoperative imaging data included (1) a lumbar spine radiograph (Figure 7) that showed lumbar degeneration without lumbar scoliosis, spondylolisthesis, or instability and (2) a CT scan in the sagittal, coronal, and axial planes showed that the distal end of the prolapsed nucleus pulposus was downward beyond the inferior margin of the L5 pedicle, implying that it was classified as type 4 according to Lee’s classification. The L5 pedicle height was 11.3 mm. MRI in the sagittal plane showed that the L4/5 nucleus pulposus migrated downward substantially (Figure 8).

**Figure 9:**
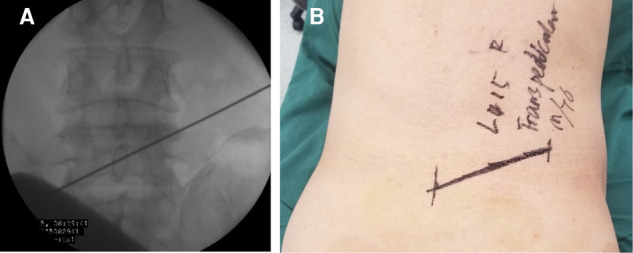
(**A**) Identification of the trajectory guided by the C-arm fluoroscopy. (**B**) The marker line is drawn, connecting from 1 to 10 o’clock on the pedicular projection.

In the prone position, the patient flexed his bilateral hip and knee joints on the operating table. Guided by the C-arm fluoroscopy, the marker line, connecting from 1 to 10 o’clock on the pedicular projection, was marked on the back skin (Figure 9). To easily distinguish the prolapsed nucleus pulposus, intervertebral disc puncture surgery under local anesthesia was performed, and 1:10 methylene blue was injected into the disc to stain the nucleus pulposus.

**Figure 10:**
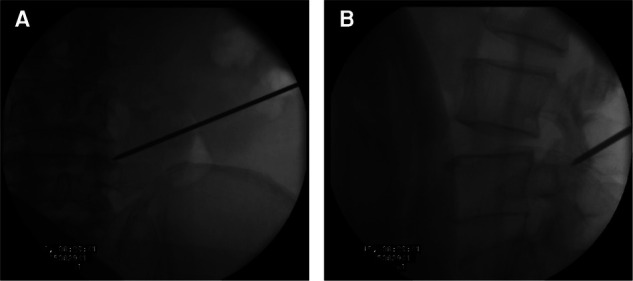
(**A**) The Kirschner wire position by the anteroposterior position of C-arm fluoroscopy. (**B**) The Kirschner wire position by the lateral position of C-arm fluoroscopy.

**Figure 11:**
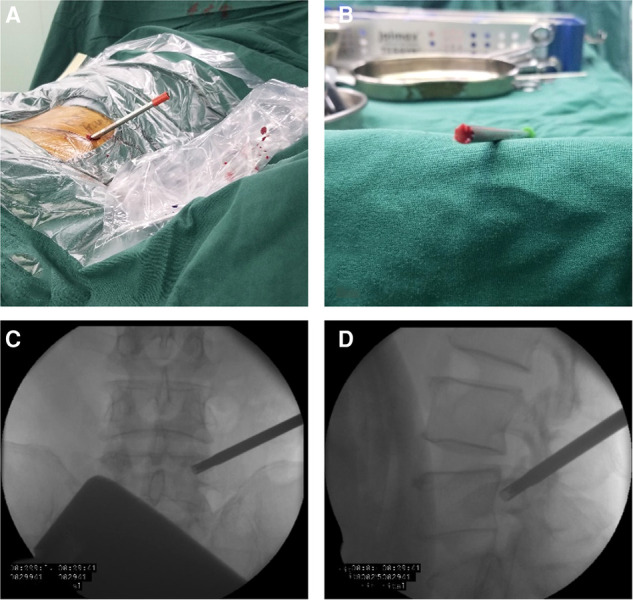
(**A**) The pedicular bone around the Kirschner wire is sawn by the trephine. (**B**) Bone can be brought out by the trephine. (**C**) The medial wall of the pedicle is the safety margin in the anteroposterior view. (**D**) The lateral view of the radiograph shows the position of the trephine.

**Figure 12:**
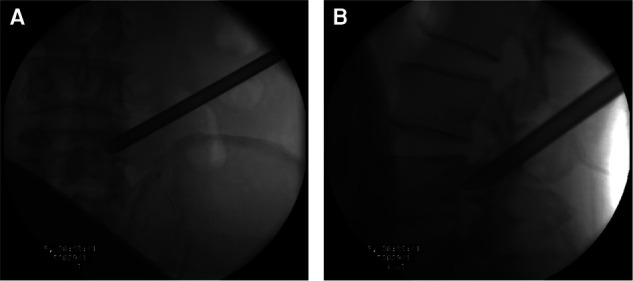
(**A**) The working tube position in the anteroposterior view. (**B**) The working tube position in the lateral view.

The Kirschner wire was advanced from the skin entry point to the target vertebral pedicle along the marker line on the skin. AP position C-arm fluoroscopic images were used to confirm the wire’s final position (Figure 10). The wire was then tapped into the pedicle with a surgical hammer. Soft tissue was progressively expanded by the dilators. The pedicular bone around the Kirschner wire was sawn by the trephine (Figure 11A). A lump of bone was extracted by the first trephine (Figure 11B). This step was monitored by C-arm fluoroscopy. The medial wall of the pedicle was the safety margin (Figures 11C,D). A working channel was established after removing the trephine (Figure 12).

**Figure 13:**
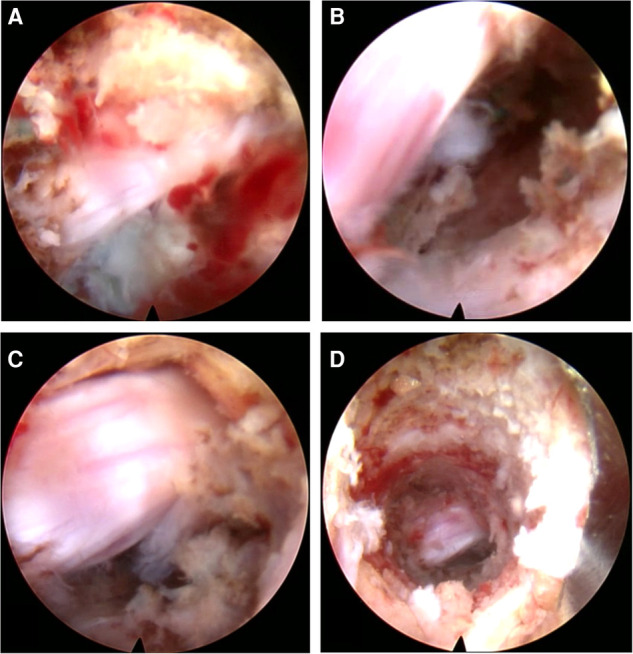
(**A**) The nerve root and the blue-staining nucleus pulposus. (**B**) The decompressed nerve root after discectomy. (**C**) The completely free nerve root. (**D**) The bone tunnel *via* the pedicle.

The L5 traversing nerve root and the prolapsed nucleus pulposus (at the ventral side of the L5 traversing nerve root) could be directly exposed under the endoscope *via* the transpedicular approach (Figure 13A). There was no yellow ligament and annulus fibrosus in this view, due to which we could expose the posterior vertebral wall and posterior longitudinal ligament after the free nucleus pulposus was removed (Figure 13B). Further exploration from distal to proximal aspect was performed to ensure that all the free nucleus pulposus was removed, and the whole traversing nerve root was not compressed (Figure 13C). After hemostasis, the bony channel in the pedicle could be observed when the endoscope was withdrawn (Figure 13D).

**Figure 14:**
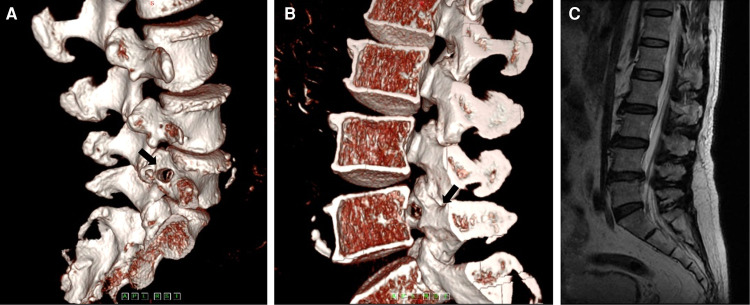
(**A**) The postoperative computed tomography shows the entrance of the bony channel on the pedicle. (**B**) The postoperative computed tomography shows the exit of the bony channel on the pedicle. (**C**) The postoperative magnetic resonance imaging shows no residual matter.

The radiation pain in the right lower extremity was rapidly relieved after surgery. The SLR test was negative. The postoperative three-dimensional CT reconstruction image showed the entrance and exit of the bony channel on the pedicle (Figures 14A,B). Bony channels at the sagittal and coronal sections and in the cross section were observed. The postoperative MRI images showed good decompression (Figure 14C).

## Discussion

Patients with a highly migrated nucleus remain challenging for endoscopic discectomy, although the technique has been widely improved in past decades. In the present case, the displaced nucleus certainly increased the complexity of the procedure because of the occlusion of the bone and nervous structures. During the surgery *via* transforaminal approach, the exiting nerve root usually opposes the manipulation of the endoscope in case of an upward-migrated herniation, and the pedicle could be the obstacle to the downward-migrated herniation.

Lee et al. (1) developed a classification based on the location of the nucleus pulposus that is displaced on the sagittal plane of the MRI and noted that the type of severe upward- and downward-migrated prolapses was not suitable for treatment with transforaminal approach endoscopic surgery due to a high failure rate, and traditional surgery was recommended. Choi et al. (10) proposed that the interlaminar approach can provide more exposure space to deal with a highly migrated disc herniation and is superior to endoscopic surgery *via* the transforaminal approach. A study (11) that compared the efficacy and safety of interlaminar endoscopic lumbar discectomy (IELD) and interlaminar microscopic lumbar discectomy (IMLD) to treat far-migrated LDH noted that both IELD and IMLD achieved favorable clinical results in the treatment of far-migrated LDH, with only minor complications. Compared with IMLD, low back pain was significantly reduced with IELD, presumably because it involved less trauma. Gizatullin et al. (12) compared the clinical outcomes after translaminar microsurgical sequestrectomy and transpedicular endoscopic surgeries and noted that the results were similar. However, postoperative back and leg pain regression, neurological recovery, and improvement in the quality of life according to the Oswestry scoring system were more common after transpedicular surgery. Meanwhile, if the interlaminar space is small, it is necessary to expand the bony window. Nevertheless, the nerve root is inevitably pulled and pushed several times during the surgery using the interlaminar approach, and some patients may have abnormal neural reactions after the surgery. In cases under local anesthesia, it is a challenge to the patients’ endurance and the surgeons’ mentality.

Krozk et al. (9) was the first researcher to handle a severely downward-migrated disc herniation through a bony channel, attempting to establish a bony channel vertically on the pedicle, in which good clinical results were achieved in the treatment of patients. This technique was later duplicated by some other researchers and similar experiments were conducted, but most of them were limited to case reports. The technique was not elaborated in detail, and long-term clinical follow-up was lacking, especially for the outcome of bony defects caused by creating the approach, which led to a lack of clarity (13–15). In a study in 2007, our team identified that for a highly migrated disc herniation, transforaminal, interlaminar, and transpedicular approaches can all be good at removing the protruding and displaced nucleus pulposus, of which the transpedicular approach is more direct, but requires good technical and equipment support (16). The method of entering the pedicle that we used in this study differed from that in the previous study with the vertical trajectory. We used a certain angle to enter the pedicle from the exterior superiorly to the interior inferiorly. The vertical entry into the pedicle to create a bone channel requires high anatomical parameters of the pedicle, and if the height of the pedicle is extremely small, the possibility of fracture and surgical failure is high. Previous research was mostly limited to European races; this interracial anatomical characteristic may not have been a consideration for previous scholars. Our cases were obtained from the Chinese population, in which the body shape and anatomical parameters varied from those of western populations. The pedicle height of the patients in this study was 11–14 mm; using a method with a certain angle to enter the pedicle could avoid this disadvantage. The lumbar segment artery travels on the side of the lumbar vertebral body, and the trephine or working cannula slides forward along the vertical trajectory; therefore, the possibility of injury to the segmental artery is extremely high. This injury can have terrible consequences, triggering retroperitoneal hemorrhage and even shock. This kind of complication, although never reported in the previous transpedicular approach discectomy, has occurred during vertebroplasty in the extra-pedicle approach with the same trajectory (17).

It is easier to enter the pedicle at a certain angle. First, the position of the bony anchor point is the deformation structure of the pedicle and the superior articular process, which has a small notch and is easily anchored by the Kirschner wire. Although the entrance we chose was on the upper pedicle, the opening in the spinal canal was still facing the position of the migrated nucleus (Figure 14). Furthermore, we could use the endoscopic 30°-angle of view to observe the proximal and distal areas and utilize some flexible tools to grasp the nucleus. During the procedure, we found that the angle between the bony tunnel and the posterior vertebral wall was the important parameter for trajectory. The angle was extremely small to explore the central region of the spinal canal. We suggest that the angle between the bony tunnel and the posterior vertebra wall should be 40°. The obvious disadvantage of this technique is that it is difficult to deal with the intervertebral space due to the limitation of the working channel in this trajectory. If necessary, it can be withdrawn from the working canal and re-entered into the intervertebral space to address the herniation through the Kambin triangle zone. In this study, we did not treat the intervertebral space again. We suggest that if the prolapsed nucleus pulposus is larger, only the discectomy in the spinal canal can be completed by the release of the nerve root. In previous studies (18), the removal of the sequestrated disc in the spinal canal and aggressive resection were compared, showing that the patient satisfaction at 2-year follow-up was higher in the limited discectomy group with a high recurrence rate. During our long-term follow-up, there was no recurrence in this group. Theoretically, the risk of recurrence might be high, because the surgery *via* the transpedicular approach cannot reach the intervertebral space. However, the fact is that most of the nucleus pulposus had prolapsed into the spinal canal and there were few residues in the intervertebral space. The number of cases in our study was small, and large samples are needed to determine the reliability of this approach in further research.

Through our regular follow-up of patients after surgery, we found that the patient’s bony channels had healed after 6 months, which was similar to the phenomenon in which the nail tunnel heals after internal fixation removal in long bone fractures, and also follows the principle of Wolff’s law.

In our opinion, precise preoperative measurement and design are essential for the transpedicular approach. A basic condition for choosing the transpedicular approach is adequate pedicle height. The oblique trajectory through the pedicle is convenient for directly finding and removing the migrated nucleus pulposus with a low risk of blood vessel injury (among pedicle and vertebral body). Care should be taken when using a trephine, and a grinding drill can be used as an assistant tool to reduce the risk of nerve root injury while establishing the bony channel. When the decompression range is found to be insufficient during the operation, the bone access can be enlarged by using drills to obtain flexible angle for decompression procedure. However, it must be noted that the use of drills to expand bone access must be performed under the guidance of the C-arm to avoid pedicle fractures.

## Conclusion

The new trajectory of the transpedicular approach with the full endoscopic technique for an extremely downward-migrated disc herniation showed excellent results in a small sample study. A precise surgical plan should be made, including measurements of pedicle height and angle of the bone tunnel.

## Data Availability

The raw data supporting the conclusions of this article will be made available by the authors, without undue reservation.
